# Estimations of Water Use Efficiency in Winter Wheat Based on Multi-Angle Remote Sensing

**DOI:** 10.3389/fpls.2021.614417

**Published:** 2021-03-30

**Authors:** Hai-Yan Zhang, Meng-Ran Liu, Zi-Heng Feng, Li Song, Xiao Li, Wan-Dai Liu, Chen-Yang Wang, Wei Feng

**Affiliations:** State Key Laboratory of Wheat and Maize Crop Science, National Engineering Research Center for Wheat, Henan Agricultural University, Zhengzhou, China

**Keywords:** winter wheat, hyperspectral remote sensing, angle adaptability, water use efficiency, monitoring model

## Abstract

Real-time non-destructive monitoring of water use efficiency (WUE) is important for screening high-yielding high-efficiency varieties and determining the rational allocation of water resources in winter wheat production. Compared with vertical observation angles, multi-angle remote sensing provides more information on mid to lower parts of the wheat canopy, thereby improving estimates of physical and chemical indicators of the entire canopy. In this study, multi-angle spectral reflectance and the WUE of the wheat canopy were obtained at different growth stages based on field experiments carried out across 4 years using three wheat varieties under different water and nitrogen fertilizer regimes. Using appropriate spectral parameters and sensitive observation angles, the quantitative relationships with wheat WUE were determined. The results revealed that backward observation angles were better than forward angles, while the common spectral parameters Lo and NDDAig were found to be closely related to WUE, although with increasing WUE, both parameters tended to become saturated. Using this data, we constructed a double-ratio vegetation index (NDDAig/FWBI), which we named the water efficiency index (WEI), reducing the impact of different test factors on the WUE monitoring model. As a result, we were able to create a unified monitoring model within an angle range of −20–10°. The equation fitting determination coefficient (*R*^2^) and root mean square error (RMSE) of the model were 0.623 and 0.406, respectively, while an independent experiment carried out to test the monitoring models confirmed that the model based on the new index was optimal, with *R*^2^, RMSE, and relative error (RE) values of 0.685, 0.473, and 11.847%, respectively. These findings suggest that the WEI is more sensitive to WUE changes than common spectral parameters, while also allowing wide-angle adaptation, which has important implications in parameter design and the configuration of satellite remote sensing and UAV sensors.

## Introduction

Wheat is one of the important food crops in the world, and with recent economic development and population growth, the level of winter wheat production has become even more important for ensuring world food security. Meanwhile, due to global climate change, lack of water has become a key limiting factor in winter wheat production. Water use efficiency (WUE) is a broad agronomic concept that reflects the comprehensive effect of crops on water use. Leaf WUE is the main criterion used to measure drought tolerance and efficient water use in crops, and subsequent selection and screening of high WUE varieties is one of the most important goals of crop breeding (Richards, 2006; Zhang et al., 2007). Efficient use of limited water resources and increases in overall WUE has therefore become an urgent goal of winter wheat production.

Leaf WUE is defined as the ratio of net photosynthesis (P_N_) to transpiration (Tr; Condon et al., 2002), which can be estimated using the carbon isotope ratio (*δ*^13^C; Hultine and Marshall, 2000). Recently, rapid development of remote sensing technology has provided an effective tool for large-scale analysis of water use in crops. Compared with traditional crop WUE monitoring and diagnostic tools, hyperspectral remote sensing technology has made it possible to obtain a huge amount of continuous large-scale data in a more efficient manner (Peñuelas et al., 1993b; Dong et al., 2011). Ground hyperspectral remote sensing technology selects sensitive bands using spectral characteristic information to obtain vegetation indexes, which are used to establish estimation models (Hatfield et al., 2008; Mistele and Schmidhalter, 2008).

An appropriate water content is the basis of vigorous plant growth and efficient water use. As early as 1971, Thomas et al. (1971) analyzed the relationship between the leaf water content (LWC) and spectral reflectance, and revealed a strong correlation with reflectance at 1450 and 1930 nm. Similarly, Carter (1991) suggested that the near-infrared absorption peak at 950–970 nm could be used to monitor plant moisture content, while Dawson et al. (1999) examined the performance of the moisture spectral index to estimate the canopy water content, revealing that indexes based on 970 and 1200 nm water absorption characteristics had a high coefficient of determination (Dawson et al., 1999). However, studies also suggest that reflectance at 970, 1200, and 1900 nm is easily affected by starch, protein, and nitrogen (Curran, 1989). For example, Sims and Gamon (2003) revealed that the best spectral bands for remote estimates of the plant water content at the canopy scale were 1150–1260 and 1520–1540 nm (Sims and Gamon, 2003), while in addition to the near infrared region, reflectance at 690 and 740 nm have also been shown to reflect water stress in plants (Dobrowski et al., 2005). Screening and analysis of spectral bands that are sensitive to water also provides a basis for the establishment of relevant vegetation indexes that reflect the water status. Peñuelas et al. (1993a) combined the water absorption band at 970 nm and the reference band at 900 nm as a ratio to establish a water index (WI) capable of tracking changes in water content. Similarly, Zarco-Tejada et al. (2003) used MODIS data to construct a plant water index (PWI) for monitoring vegetation moisture content, and revealed good consistency with the water content of ground crops (Zarco-Tejada et al., 2003). Moreover, the floating-position water band index (FWBI) has also been established, which uses the reflectance at 900 nm and minimum reflectance at 900–980 nm to represent the water status (Strachan et al., 2002). Yao et al. (2014) subsequently introduced a new water-sensitive band based on the normalized vegetation index NDSI (1429, 416) to construct a three-band vegetation index capable of estimating the leaf equivalent water thickness. However, these previous studies mainly used the sensor to obtain two-dimensional information of the crop in a vertical direction, and failed to include data from middle to lower parts of the canopy. The accuracy of remote sensing monitoring therefore requires further improvements.

Compared with vertical observation angles, the multi-angle observation method collects data from different directions, providing multi-dimensional information and representing a new method of remote sensing monitoring (Thenkabail et al., 2000; Pocewicz et al., 2007; Huang et al., 2011). A number of studies have been carried out to extract optical and structural information using multi-angle observations (Cierniewski et al., 2004; Rautiainen et al., 2004), suggesting that multi-angle remote sensing technology can improve the ability of a vegetation index to estimate crop canopy structure and distinguish between crop varieties (Shibayama and Wiegand, 1985; Diner et al., 1999). The photochemical reflectance index (PRI) is notably affected by the observation angle. For example, PRI values calculated using backward spectral data tend to be higher than those obtained with forward observation data (Drolet et al., 2008; Garbulsky et al., 2011; Middleton et al., 2011). Furthermore, Galvão et al. (2009) found that data collected in a backscattering direction was better at distinguishing between different soybean varieties, while Chopping et al. (2003) obtained canopy characteristics of desert grassland using multi-angle remote sensing data. Chen et al. (2005) and Leblanc et al. (2005) proposed a multi-angle index for measuring leaf aggregation based on hot and dark spot reflectivity. Multi-angle hyperspectral remote sensing has also made great progress in estimations of crop pigment content and nitrogen content (Stagakis et al., 2010; He et al., 2015). Meanwhile, He et al. (2016) constructed an angle insensitivity index (AIVI) based on analysis of different bands and vegetation indexes, improving the accuracy of plant nitrogen content estimations and expanding the scope of application.

In the field of remote sensing monitoring, in addition to analyses of vegetation canopy structure and physiological indicators, the performance indicators of crop production are also important. The P_N_ of field crops has been shown to be significantly correlated with physiological indicators, and studies suggest the use of the ratio vegetation index (R_810/680_) to directly estimate the P_N_ of rice leaves (Tian et al., 2005). In addition, based on hyperspectral data, Zhang et al. (2018) established an estimation model of nitrogen fertilizer use efficiency in winter wheat, while photosynthetic effective radiation (FPAR) captured by the canopy can also be directly obtained through multi-angle remote sensing (Chen et al., 2003).

Light use efficiency (LUE) is one of the most important traits in crops, and is usually reflected by the PRI (Zarco-Tejada et al., 2013). For example, Hall et al. (2008) proposed a method to obtain forest LUE directly from space by measuring the shadow component of the PRI based on multi-angle spectral information (Hall et al., 2008). Meanwhile, Bastiaanssen et al. (1999) used remote sensing data to estimate crop yield and crop transpiration (ETc), and then determined the WUE of crops in the Barkra region of India. Li et al. (2005) also used remote sensing observation means combined with meteorological data to invert crop WUE in the Haihe River Basin by estimating crop transpiration. Thus, while progress has been made in the use of remote sensing data to monitor crop WUE, the monitoring indicators, methods, and models remain inconsistent due to geographical differences and crop types, as well as differences in cultivation conditions. However, estimations of WUE utilization efficiency at the leaf scale based on hyperspectral remote sensing data are lacking, especially with regards the influence of different observation angles, and the angle range of model adaptation requires further clarification. The main goal of this study, therefore, was to create a model capable of estimating the instantaneous WUE of winter wheat leaves based on multi-angle hyperspectral remote sensing data. By clarifying the relationship between common spectral parameters and WUE at different vertical angles, a new vegetation index for estimating WUE was established. The new parameter was then compared with common vegetation indexes under different observation angles, and the optimal range of angles was determined, allowing establishment of a unified estimation equation. The findings provide a theoretical basis for real-time accurate monitoring of water use in winter wheat, supporting the screening of germplasm resources and efficient irrigation management.

## Materials and Methods

### Experimental Design

Five experiments were carried out across 4 years at two different locations. Various water management, N rates, and cultivars of hexaploid winter wheat (*Triticum aestivum* L.) were studied, specific details are shown in Table 1. Experiments 1–4 were completed in the experimental station of Henan Agricultural University (35°51’N, 113°35’S), Zhengzhou, Henan Province, China, in fluvo-aquic soil. Experiments 1 and 4 were completed in 2016–17, and experiments 2 and 3 in 2017–18 and 2018–19, respectively. Experiment 5 was completed at Shangshui experimental station in Zhoukou, Henan Province (33°33’N, 114°37’S), in 2017–18, in lime concretion black soil. The experiments 1, 4, and 5 consisted of a only one irrigation regiments (twice irrigation, 750 m^3^ha^−1^ at jointing plus anthesis stage), experiments 2–3 consisted of a three irrigation regiments (no irrigation, single irrigation of 750 m^3^ha^−1^, and irrigation750 m^3^ha^−1^ at jointing plus anthesis stage). Three different winter wheat cultivars were examined, two erect (Yumai 49–198 and Zhoumai 27) and one horizontal (Zhengmai 9694). Experimental plots 1–4 covered an area of 7 × 2.9 m, respectively, planted in a north-south direction, with 18 cm row spacing, while plot 5 covered an area of 9 × 6 m, planted in a north-south direction, with 20 cm row spacing. All experiments followed a completely randomized block design, and each treatment was repeated three times. All plots were managed according to local standard management practices.

**Table 1:** Seasons, soil status, cultivars, nitrogen rates, irrigation frequency, and sampling dates for five experiments.

Exp. no.	Season, Site, and Cultivar	Soil characteristics	Treatments	Sampling stage
Exp. 1	2016-2017 Zhengzhou Yumai49-198	Type: fluvo-aquic soil, Organic-M: 20.7 g kg^−1^, Soil pH (CaCl_2_): 7.9, Total N: 1.9 g kg^−1^,AvailableP: 40.63 mg kg^−1^, Available K: 116.2 mg kg^−1^	Irrigated N: N rate (kg ha^-1^), W_2_: [N_0_(0), N_6_(60), N_12_(120), N_18_(180), N_24_(240)]. N: 50% prior to seeding and 50% at jointing. Irrigation frequencies: W_2_ (twice at jointing and anthesis stage).	Booting
				Anthesis
				Mid-filling
Exp. 2	2017-2018 Zhengzhou Yumai49-198	Type: fluvo-aquic soil, Organic-M: 16.8 kg^−1^, Soil pH (CaCl_2_): 7.8, Total N: 0.92 g kg^−1^, Available P: 18.90 mg kg^−1^, Available K: 152.64 mg kg^−1^	Water and nitrogen coupling: N rate (kg ha^-1^), W_0_: [N_0_(0), N_6_(60), N_12_(120), N_18_(180), N_24_(240)], W_1_: [N_0_(0), N_6_(60), N_12_(120), N_18_(180), N_24_(240)], W_2_: [N_0_(0), N_6_(60), N_12_(120), N_18_(180), N_24_(240)]. Irrigation frequencies: W_0_(none), W_1_(once at jointing stage), W_2_ (twice at jointing and anthesis stage).	Booting
				Heading
				Anthesis
				Mid-filling
Exp. 3	2018-2019 Zhengzhou Yumai49-198	Type: fluvo-aquic soil, Organic-M: 16.8 kg^−1^, Soil pH (CaCl_2_): 7.8, Total N: 0.92 g kg^−1^, Available P: 18.90 mg kg^−1^, Available K: 152.64 mg kg^−1^	Water and nitrogen coupling: N rate (kg ha^-1^), W_0_: [N_0_(0), N_6_(60), N_12_(120), N_18_(180), N_24_(240)], W_1_: [N_0_(0), N_6_(60), N_12_(120), N_18_(180), N_24_(240)], W_2_: [N_0_(0), N_6_(60), N_12_(120), N_18_(180), N_24_(240)]. Irrigation frequencies: W_0_(none), W_1_(once at jointing stage), W_2_ (twice at jointing and anthesis stage).	Booting
				Heading
				Anthesis
				Mid-filling
Exp. 4	2016-2017 Zhengzhou Zhengmai9694	Type: fluvo-aquic soil, Organic-M: 16.8 kg^−1^, Soil pH (CaCl_2_): 7.8, Total N: 0.92 g kg^−1^, Available P: 18.90 mg kg^−1^, Available K: 152.64 mg kg^−1^	Irrigated N: N rate (kg ha-1), W_2_: [N_0_(0), N_12_(120), N_18_(180), N_24_(240)]. N: 50% prior to seeding and 50% at jointing. Irrigation frequencies: W_2_ (twice at jointing and anthesis stage).	Booting
				Heading
				Anthesis
				Mid-filling
Exp. 5	2017-2018 Shangshui Zhoumai27	Type: lime concretion black soil, Organic-M: kg^−1^, Soil pH (CaCl2): 7.0, Total N: kg^−1^, Available P: 4.87 mg kg^−1^, Available K: 176.52 mg kg^−1^	Irrigated N: N rate (kg ha-1), W_2_: [N_0_(0), N_6_(60), N_12_(120), N_18_(180), N_24_(240)]. N: 50% prior to seeding and 50% at jointing. Irrigation frequencies: W_2_ (twice at jointing and anthesis stage).	Heading
				Anthesis

### Measurements of Agronomic Indicators

The P_N_ and Tr of the top leaf were determined in the field using a photosynthetic device (LI-6400 photosynthetic rate system; Li-Cor Inc., United States). Measurements were obtained in the open at a carbon dioxide concentration of approximately 385 μmol 1^−1^. The built-in light source was set at 1600 μmol m^−2^ s^−1^. The ratio of P_N_ and Tr was then used to reflect the WUE. Measurements were taken at the booting, heading, anthesis, initial-filling, and mid-filling stages.

Twenty representative plants from each treatment were then randomly selected and brought back to the laboratory where they were separated into stem and leaf samples. Leaf weight (FW) was recorded before drying the samples in an oven at 105°C for 30 min then to a constant weight at 70°C. The dry mass of the leaves (DW) was then determined and the LWC was calculated as follows:

LWC = (FW-DW)/FW.

Plant leaf samples were simultaneously dried to a constant weight then crushed and passed through a sieve before determining the leaf nitrogen content (LNC) using the Kjeldahl method.

### Canopy Spectrum Acquisition

**Figure 1:**
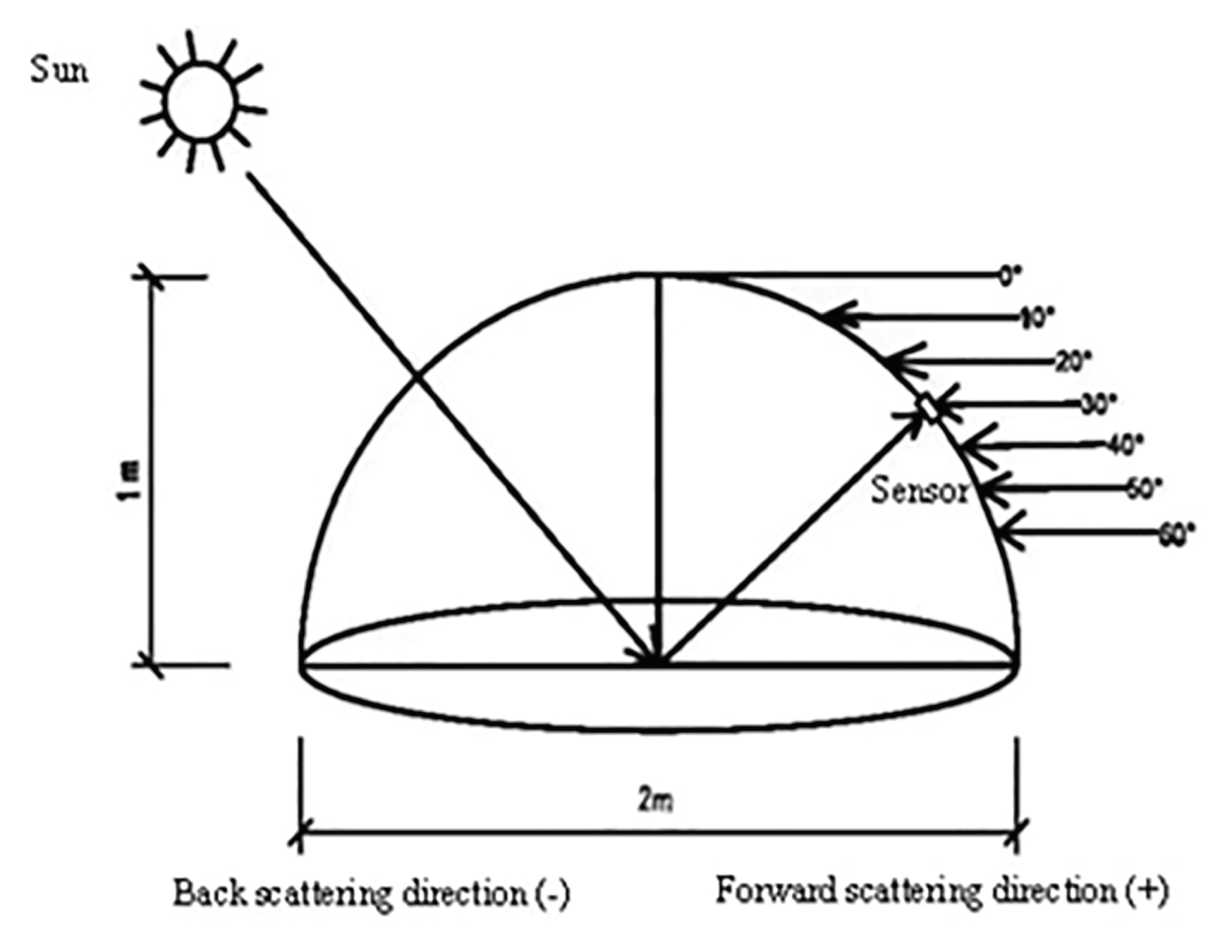
Dimensions and design of the field goniometer system.

At the same time as measuring the WUE, the spectral reflectance of the winter wheat canopy was also determined. A FieldSpec Pro FR 2500 back-mounted field hyperspectral radiometer (Analytical Spectral Device, American ASD Company) was used to sample 10 points per 1 m^2^, which were then averaged as one point of data. Measurements were made on a sunny day with no cloud cover between 10:00 and 12:00 a.m. The field of view of the spectrometer was set at 25°, the spectral range was 350–1075 nm, and the sampling interval was 1.6 nm. Before sampling and during use, a 40 × 40 cm BaSO_4_ whiteboard was used for calibration. To obtain multi-angle spectrum, a probe was fixed to the multi-angle observation frame according to the design of the field angle measurement system (FIGOS, Figure 1). A total of 13 observation angles were examined following the principal plane of the sun, with the sunny side representing backward observation angles (−60, −50, −40, −30, −20, and −10°, respectively), and angles on the opposite side representing forward observation angles (10, 20, 30, 40, 50, and 60°, respectively), with the vertical angle set at 0°.

### Data Application

A self-developed computation program was used to optimize the sensitive band combinations and equations using MATLAB 7.0 software. Data from experiments 1–3 were used to construct the new vegetation index and WUE estimation model, while independent data from experiments 4–5 were used to test the model by comparing differences between the coefficient of determination (*R*^2^), root mean square error (RMSE), and relative error (RE, %). A 1:1 scatter plot was then used to show the effect of the model. Some common spectral indices were calculated using the equation listed in Table 2. RMSE and RE were calculated as follows:

(1) $$\mathrm{RMSE}=\sqrt{\frac{1}{n}\times \overset{n}{\underset{i=1}{\sum }}{\left(P_{i}-Q_{i}\right)}^{2}}$$

(2) $$\mathrm{RE}=\sqrt{\frac{1}{n}\times \overset{n}{\underset{i=1}{\sum }}{\left(\frac{P_{i}-Q_{i}}{Q_{i}}\right)}^{2}}\times 100$$

where P_i_ and Q_i_ represent the predicted and measured values, respectively, and n represents the number of samples.

## Results

### Quantitative Relationships Between the Leaf Nitrogen Content, Water Content, and WUE

**Figure 2:**
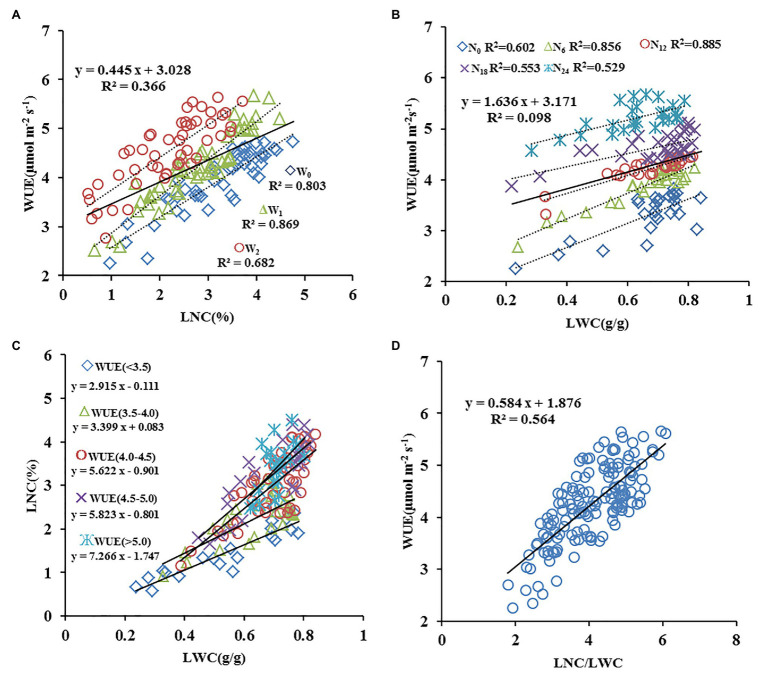
Quantitative relationships between leaf nitrogen (N) content (LNC: A), water content (LWC: B), LNC/LWC (D) and water use efficiency (C: The relation between LWC and LNC. WUE; *n* = 140).

Based on the data from experiments 1–3, the relationships between the LNC, LWC, and ratio between LNC/LWC under different experimental conditions was analyzed in terms of the WUE (Figure 2). As shown in Figure 2A, when the irrigation treatment conditions are not distinguished, the relationship between LNC and WUE was generally poor (*R*^2^ = 0.366). Under a single water treatment condition, the LNC showed a significant linear relationship with the WUE, and the correlation was best under W_1_ (once water at jointing stage) conditions (*R*^2^ = 0.869), followed by W_0_ conditions (*R*^2^ = 0.803). The worst correlation was observed under W_2_ (twice water at jointing and anthesis stage) conditions (*R*^2^ = 0.682). Similarly, the relationship between the LWC and WUE was also affected by nitrogen treatment. Overall, the *R*^2^ between the LWC and WUE was only 0.098 (Figure 2B). Under different nitrogen treatment conditions, the relationship between LWC content and WUE was significant. The transition from high to low nitrogen treatment caused an increase followed by a decrease in *R*^2^, with N_12_ (120 kg ha^−1^) treatment giving the highest value (*R*^2^ = 0.885). The relationships between the LNC and LWC content and WUE were therefore affected by each other, and these relationships were therefore analyzed further. Results revealed that the variation in WUE was closely related to the slope between LNC and LWC. With increasing WUE, the slope of the equation between LNC and LWC gradually increased (Figure 2C), and there was a significant positive correlation between the LNC/LWC ratio and WUE (*R*^2^ = 0.564, Figure 2D). These findings suggest that the LNC/LWC ratio more accurately reflects the dynamic changes in WUE under different water and nitrogen conditions.

**Table 2:** Summary of selected spectral parameters reported in the literature.

Vegetation indices	Formula	Reference
DVI(810,680)	R_810_-R_680_	Jordan, 1969
SRPI	R_430_/R_680_	Peñuelas et al., 1995a
WBI-1	R_950_/R_900_	Peñuelas et al., 1993a
WI	R_900_/R_970_	Peñuelas et al., 1997
Readone	R_415_/R_695_	Read et al., 2002
Lo	min(R_680-780_)	Miller et al., 1990
PSRI	(R_680_-R_500_)/R_750_	Merzlyak et al., 1999
R_434_/(R_496_ + R_401_)	R_434_/(R_496_ + R_401_)	Tian et al., 2011
R_705_/(R_717_ + R_491_)	R_705_/(R_717_ + R_491_)	Tian et al., 2011
FWBI	R_900_/min(R_930-980_)	Strachan et al., 2002
PRI(570, 531)	(R_531_-R_570_)/(R_531_ + R_570_)	Gamon et al., 1992
SIPI(800, 680, 445)	(R_800_-R_445_)/(R_800_-R_680_)	Peñuelas et al., 1995a
mSR705	(R_750_-R_445_)/(R_705_-R_445_)	Sims and Gamon, 2002
RES	(R_718_-R_675_)/(R_755_-R_675_)	Ju et al., 2010
NDVI(895, 675)	(R_895_-R_675_)/(R_895_ + R_675_)	Santos and Negri, 1997
NDRE	(R_790_-R_720_)/(R_790_ + R_720_)	Fitzgerald et al., 2006
NRI(570, 670)	(R_570_-R_670_)/(R_570_ + R_670_)	Li et al., 2005
RDVI(800, 670)	(R_800_-R_670_)/sqrt(R_800_ + R_670_)	Roujean and Breon, 1995
NDDAig	(R_755_ + R_680_−2 × R_705_)/(R_755_−R_680_)	Feng et al., 2014
NDGI	[R(_520-560_)-R(_630-690_)]/ [R(_520-560_) + R(_630-690_)]	Rouse et al., 1974
EVI-1	2.5*(R_860_-R_645_)/(1 + R_860_ + 6*R_645_-7.5*R_470_)	Huete et al., 2002
MCARI(700, 670, 550)	[(R_700_-R_670_)-0.2*(R_700_-R_550_)]*(R_700_/R_670_)	Daughtry et al., 2000
Vari-GREEN	(R_520-560_-R_630-690_)/(R_520-560_ + R_630-690_-R_430-470_)	Gitelson et al., 2002
TSAVI(800, 670)	1.4735*(R_780_ + 1.4735*R_650_-1.3681)/(-1.4735*R_780_+R_650_ + 1.4735*1.3681)	Baret and Guyot, 1991
WEI	[(R_755_+R_680_-2*R_705_)*Min(R_930-980_)]/[(R_755_-R_680_)*R_900_]	This study

### Relationship Between Common Spectral Parameters and WUE at the Vertical Observation Angle

**Figure 3:**
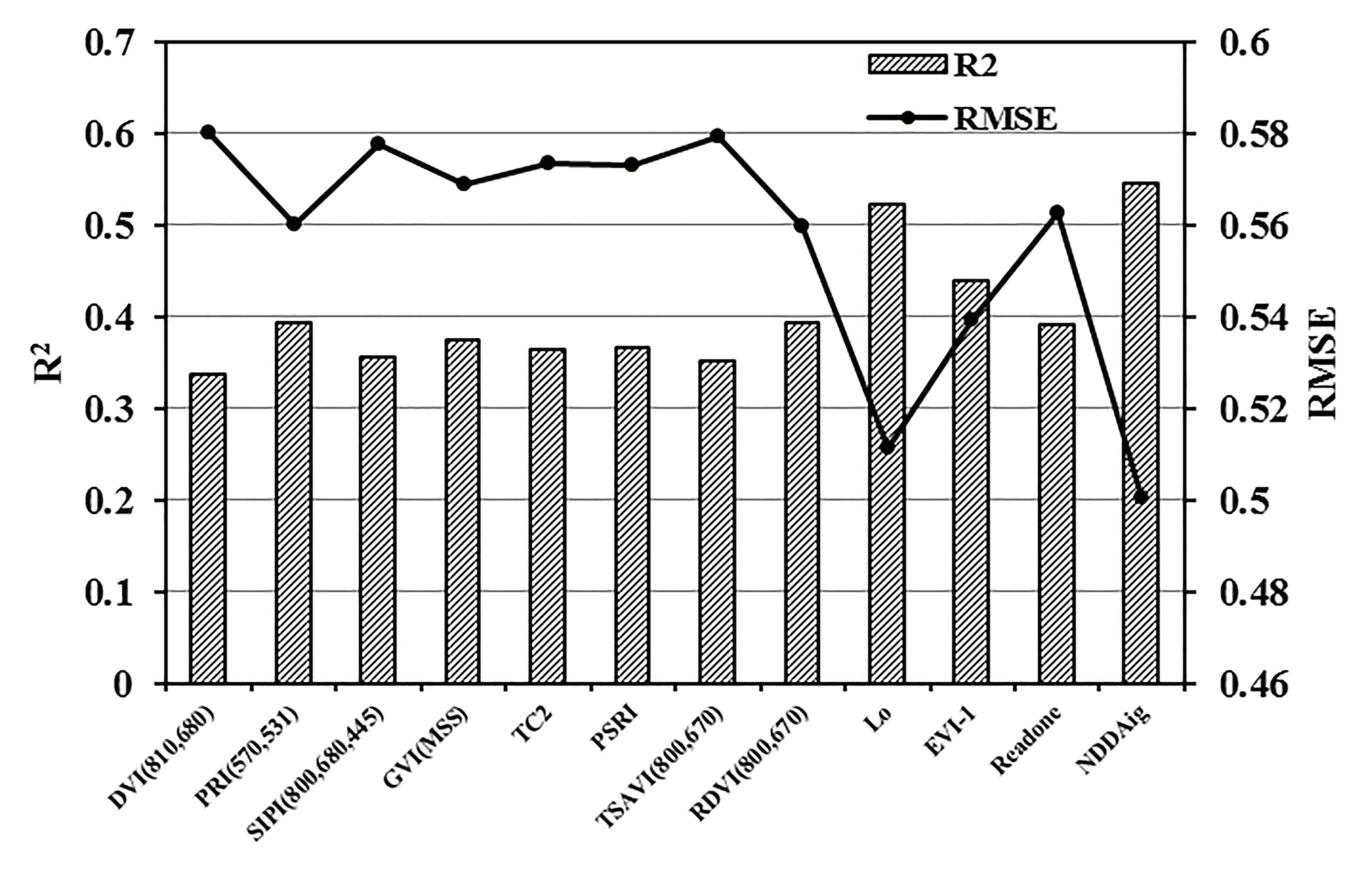
Relationships between common vegetation indices and WUE (*n* = 140).

**Figure 4:**
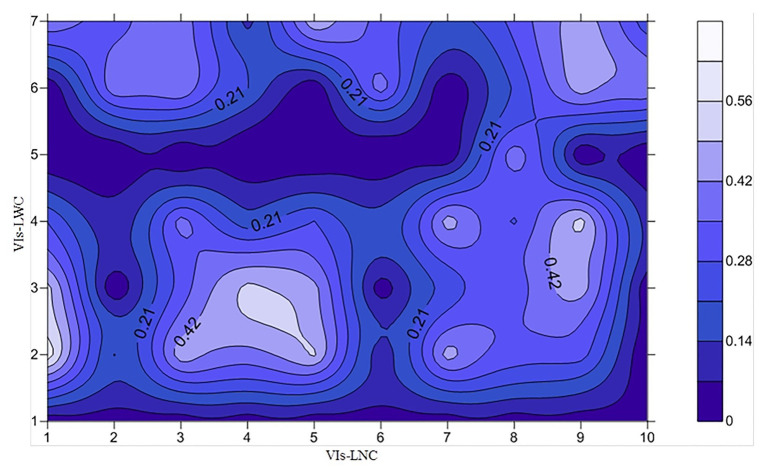
Correlations between different parameter ratios and the WUE. [X-axis: VIs-LNC (vegetation indices related to LNC) 1–10 represent NDDAigig, R_434_/(R_496_ + R_401_), R_705_/(R_717_ + R_491_), SRPI, NDRE, Lo, NRI, NDGI, RES, MCART (700, 670, and 550), respectively; Y-axis: VIs-LWC (vegetation indices related to LNC) 1–7 represent PRI (570 and 531), FWBI, WBI-1, WI, Vari-GREEN, mSR705, NDVI (895 and 675), respectively; *n* = 140].

The relationships between 330 previously reported vegetation indices and WUE were subsequently analyzed, then the best 12 were selected (Figure 3). As shown in the figure, the *R*^2^ between WUE and only three of these parameters was greater than 0.4 (*R*^2^ of Lo, EVI-1 and NDDAig: 0.439, 0.523, and 0.545; RMSE: 0.539, 0.511, and 0.500, respectively). To further improve the estimation accuracy using the relationship between LNC/LWC and WUE, LNC and LWC were converted using related vegetation indexes then the relationship with WUE was analyzed further. Ten indexes representing the LNC and seven representing the LWC were combined to create different ratios of the two vegetation indexes then the correlations with WUE were determined at a vertical angle (Figure 4). Ten combinations showed a *R*^2^ greater than 0.40, and of these, three had an *R*^2^ exceeding 0.50 and were considered optimal (NDDAig/FWBI, NDDAig/WBI-1, and SPRI/WBI-1). Overall, the combination of NDDAig/FWBI performed the best (*R*^2^ = 0.624).

**Figure 5:**
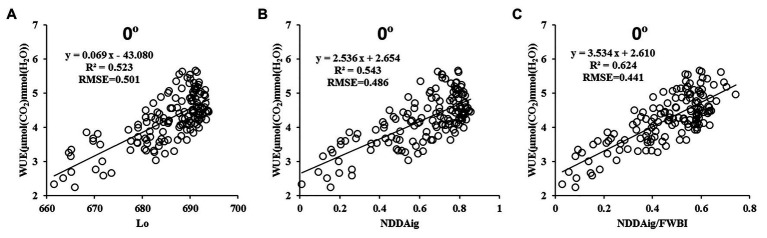
Relationships between Lo (A), NDDAig (B), NDDAig/FWBI (C), and the WUE at a 0° zenith angle (*n* = 140).

The quantitative relationships between the two common spectral parameters Lo and NDDAig and the optimized novel ratio vegetation index (NDDAig/FWBI) and WUE are shown as a scatter plot in Figure 5. A significant linear correlation was revealed between Lo and WUE (*R*^2^ = 0.523 and RMSE = 0.501, Figure 5A), with an obvious saturation phenomenon. Compared with Lo, the NDDAig model showed improvement (*R*^2^ = 0.543, RMSE = 0.486, Figure 5B), although the combined NDDAig/FWBI index gave the best results (*R*^2^ = 0.624, 19.31 and 14.92% higher than that of Lo and NDDAig, respectively; RMSE = 0.441, 13.61, and 10.20% lower than that of Lo and NDDAig, respectively), with obvious weakening of the saturation phenomenon. Based on highest *R*^2^ values, the new ratio vegetation index (NDDAig/FWBI) was constructed to generate a water efficiency index (WEI).

### Relationships Between the Spectral Parameters and WUE at Different Zenith Angles

Based on the data from experiments1–3, the relationships between the 12 common vegetation indexes and new combined index WEI were analyzed in terms of WUE under different observation angles (Table 3). Overall, except for angles of −60 and −50°, all spectral parameters had a higher backward *R*^2^ than forward *R*^2^, especially in the range of −40–30°. Moreover, the optimal observation angles of the different vegetation indexes were inconsistent with the monitoring accuracy. Two of the 13 spectral parameters (GVI and TC_2_) had an optimal observation angle of 0° with *R*^2^ values of 0.375 and 0.365, respectively, while five spectral parameters [TSAVI (800, 670), RDVI (800, 670), PSRI, SIPI (800, 680, 445), and EVI-1] had an optimal angle of −20° with *R*^2^ values of 0.424, 0.406, 0.407, 0.422, and 0.472, respectively. It is worth noting that the optimal observation angle of seven of the indexes was −10°, suggesting that observation angles of −10 to −20° are important when monitoring the WUE of winter wheat leaves.

**Table 3:** Coefficients of determination (*R*^2^) of the linear relationships between leaf water use efficiency and the vegetation indices from different view zenith angles.

	–60º	–50º	–40º	–30º	–20º	–10º	0º	10º	20º	30º	40º	50º	60º
DVI(810,680)	0.246	0.322	0.336	0.316	0.342	0.351	0.337	0.334	0.306	0.285	0.263	0.237	0.237
PRI(570,531)	0.265	0.326	0.351	0.392	0.403	0.405	0.394	0.372	0.342	0.339	0.323	0.325	0.298
SIPI(800,680,445)	0.278	0.346	0.391	0.41	0.422	0.394	0.357	0.330	0.320	0.320	0.305	0.301	0.233
GVI(MSS)	0.244	0.324	0.344	0.317	0.346	0.359	0.375	0.355	0.315	0.287	0.263	0.228	0.231
TC_2_	0.227	0.304	0.324	0.296	0.328	0.343	0.365	0.348	0.305	0.277	0.253	0.216	0.218
PSRI	0.251	0.311	0.346	0.392	0.407	0.393	0.366	0.346	0.321	0.314	0.293	0.283	0.229
TSAVI(800,670)	0.295	0.404	0.438	0.435	0.424	0.392	0.353	0.323	0.308	0.302	0.299	0.309	0.285
RDVI(800,670)	0.310	0.397	0.411	0.401	0.406	0.403	0.395	0.373	0.348	0.33	0.315	0.301	0.299
Lo	0.398	0.461	0.491	0.492	0.506	0.523	0.523	0.509	0.494	0.492	0.492	0.471	0.442
EVI-1	0.368	0.437	0.469	0.48	0.472	0.455	0.439	0.418	0.414	0.415	0.424	0.435	0.333
Readone	0.221	0.288	0.331	0.373	0.397	0.416	0.39	0.399	0.398	0.402	0.431	0.368	0.217
NDDAig	0.459	0.507	0.521	0.526	0.541	0.548	0.545	0.535	0.532	0.522	0.514	0.51	0.497
WEI	0.485	0.551	0.587	0.594	0.616	0.635	0.624	0.613	0.596	0.582	0.578	0.572	0.554
Average	0.295	0.366	0.392	0.398	0.411	0.416	0.407	0.387	0.367	0.356	0.348	0.331	0.298

**Figure 6:**
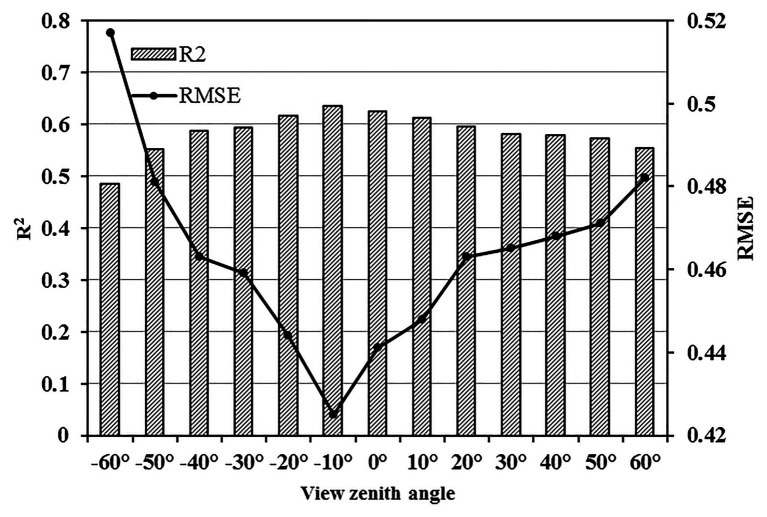
Correlations between water efficiency index (WEI) and WUE at different zenith angles (*n* = 140).

Compared with the common spectral parameters, the new vegetation index WEI showed obvious advantages at specific observation angles, especially an angle of −10°. The *R*^2^ and RMSE of the WEI model at different zenith angles are shown in Figure 6. The *R*^2^ was highest in an angle range of −20–10° and the RMSE was relatively low.

### Relationship Between the New Spectral Parameter and WUE Under Different Angle Ranges

**Figure 7:**
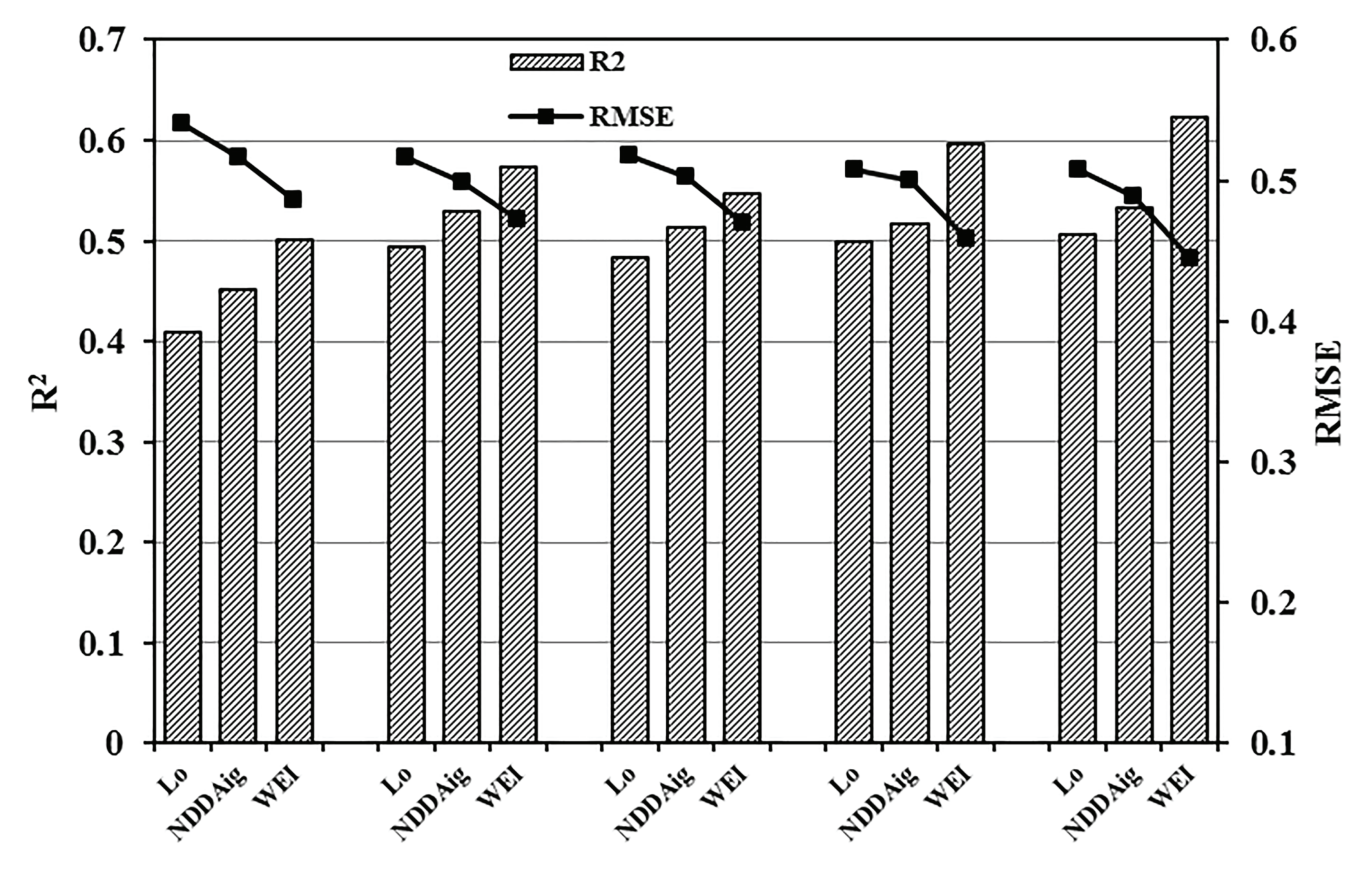
Comparisons of the predictive abilities of Lo, NDDAig, and WEI within five zenith angle ranges (−60–60°, −60–0°, 0–60°, −20–20°, and −20–10°) with respect to WUE (*n* = 140).

**Figure 8:**
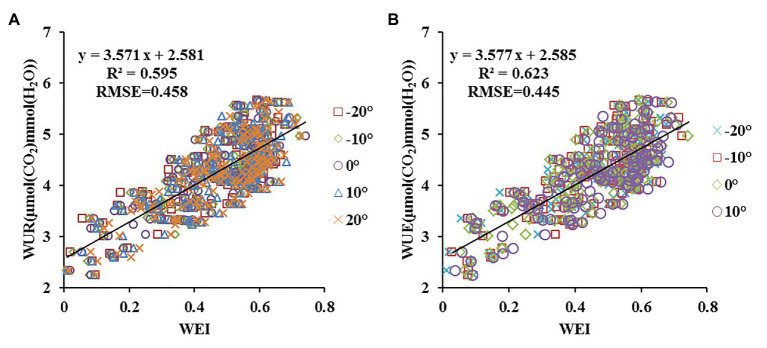
Comparisons of the predictive power of WEI at different view zenith angles (VZAs) combinations in terms of WUE (A: *n* = 700; B: *n* = 560).

From a single observation angle point of view, the monitoring accuracy of the new combined index was highest within a range of −20–10°, with highest precision at −10° (*R*^2^ and RMSE: 0.635 and 0.441, respectively). By combining the data from different observation angles, equation fitting was further analyzed under five observation angle ranges according to the principle of adjacent observation angles. As shown in Figure 7, the monitoring accuracy of WEI was higher than that of Lo and NDDAig, and the RMSE value was lowest under different angle ranges. Compared with an observation angle of −10°, the *R*^2^ of WEI decreased by 6.72% within a range of −20–20°, while the RMSE increased by 7.76% (Figure 8A). Meanwhile, the *R*^2^ decreased by only 1.93% in a range of −20–10° and the RMSE increased by only 4.71% (Figure 8B). These findings suggest that within an angle range of −20–10°, the WEI model reduces the dependency on the observation angle, increasing the applicability and stability of the model.

### Testing of the Estimation Model

**Figure 9:**
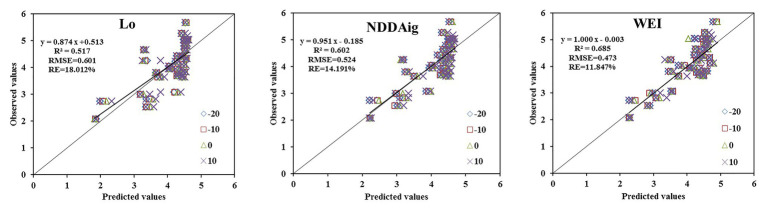
Comparisons between predicted and measured WUE based on Lo, NDDAig, and WEI at a zenith angle of −20° to +10° (*n* = 120).

The WUE estimation models were subsequently tested with the independent test data obtained in experiments 4–5 using three indicators (*R*^2^, RMSE, and RE). The prediction ability is shown as the ratio between predicted and observed values in Figure 9. The WUE model using NDDAig (*R*^2^ = 0.602, RMSE = 0.474, and RE = 14.191%) as a variable was better than that using Lo (0.517, 0.523, and 18.012%, respectively); however, new parameter WEI gave better predictions, with *R*^2^ = 0.685, RMSE = 0.403, and RE = 11.147%. Overall, these findings suggest that the new combined index of WEI can be used to accurately monitor the WUE of winter wheat leaves.

## Discussion

Water and nutrients are not only the main stress factors affecting agricultural production, but they also interact with each other, playing individual as well as complimentary roles. The main aim of agricultural management, therefore, is to maximize the coupling effect of water and nitrogen, adjust water management according to nitrogen absorption, and use water management to promote nitrogen absorption (Sheshbahreh et al., 2019). Water stress significantly affects nitrogen absorption, while improved soil water conditions benefit nitrogen absorption and utilization. Increased uptake of nutrients under drought stress can also help improve drought resistance, while optimal increases in nitrogen fertilizer application can improve WUE and increase yield (Wolfe et al., 1988). As a result of the synergetic relationship between soil water and nitrogen, the changes in crop nitrogen and water contents are also synchronized. However, this relationship is also affected by irrigation and nitrogen fertilizer treatment. Meanwhile, this study confirmed that the relationship between the LNC and WUE is also affected by irrigation treatment, while at the same time, the relationship between the LWC and WUE is also affected by nitrogen fertilizer treatment. Thus, the use of LNC and LWC alone was relatively unreliable in characterizing the WUE. In contrast, the relationship between the ratio of LNC/LWC in terms of WUE was relatively less affected by irrigation and nitrogen fertilizer treatment. The coefficient of determination of the fitting equation was 0.564, suggesting that the LNC/LWC ratio is a good indicator of dynamic changes in WUE. Overall, the findings confirmed that an increase in nitrogen in line with an increase in the water content of crop leaves is beneficial to overall water absorption.

The WUE of a plant is genetically controlled as well as being affected by the environment, and can therefore be improved by both breeding and cultivation measures. A high WUE is beneficial in maintaining a certain yield under water stress, and therefore has important application value. In addition, WUE plays a significant role in estimations of net primary productivity (NPP = WUE × Tr) on a regional scale. However, recent studies have shown that the WUE is not constant, but rather it varies greatly with environmental conditions and the plant type (Yu et al., 2001). The use of remote sensing to rapidly and non-destructively determine the real-time WUE of a crop therefore provides important information for terrestrial ecosystem and water cycle models at different scales. The crop canopy spectrum provides mixed information, and is susceptible to factors such as plant coverage, soil type, and leaf area. Accordingly, a number of studies have aimed to construct new indexes that reduce noise and improve the estimation accuracy (Wang et al., 2012). For example, Peñuelas et al. (1997) and Pinol et al. (1998) successfully determined the humidity of combustibles using spectral data obtained in the field in the Mediterranean using a combined ratio spectral index (Peñuelas et al., 1997; Pinol et al., 1998). Studies have also shown that double-ratio vegetation indexes can reduce the effects of variables such as background and leaf area index (LAI), and provide more useful information for estimations of the vegetation canopy water content (Daughtry et al., 2000; Haboudane et al., 2002). Meanwhile, double-ratio vegetation indexes also include more sensitive bands, thus improving the estimation accuracy (Gitelson et al., 2017). Based on these studies, we therefore combined the close relationship between LNC/LWC and WUE to obtain a new spectral parameter, one indicate the change in nitrogen content of the leaf is selected, another parameter that is sensitive to the change in LWC is selected, the combination of these two vegetation indexes in the form of ratio provides an opportunity to invert WUE of leaves. To this end, we selected two spectral parameters of nitrogen content (NDDAig) and water content (FWBI), and combined the two (NDDAig/FWBI) to give a new index, WEI. Accordingly, estimations of the WUE of winter wheat leaves were greatly improved. Compared with common spectral parameters, the new WEI performed best at 13 angles, with optimal angle compatibility within the range of −20–10° (*R*^2^ = 0.623). The use of independent test data also confirmed the accuracy of the model.

The difference between a vegetation index in different observation directions depends on various factors, including the crop canopy structure, the shape, and angle of the sensor, shadows, and the soil type (Kimes et al., 1985). In this study, the relationship between WEI and WUE also varied depending on the angle of observation. Pocewicz et al. (2007) made full use of the hotspot effect of the backward observation angles to improve the estimation accuracy of LAI (Pocewicz et al., 2007). Meanwhile, a multi-angle observation method was used to collect image data of the soybean canopy at different growth stages in the field, revealing that a 40° zenith angle was the best angle for inverting chlorophyll density (Zhang et al., 2013). In this study, the *R*^2^ between the spectral parameters and WUE decreased with increasing observation angle, possibly because a smaller angle results in more comprehensive information of the upper, middle, and lower canopies of the entire crop. The absorption of water by crop leaves is the result of interactions throughout the canopy; therefore, small-angle spectral information is important in determining an accurate WUE. In addition, the effects of backward observation angles were found to be better than those of forward observations. This is thought to be because when data is collected in a backward direction, the sensor is located on the same side as the sun, and data is mainly collected from canopy falling within the light. In contrast, the crop canopy with a larger shadow share is collected in a forward direction, although angles of −60° and −50° behave abnormally, possibly due to the decline in data quality at larger angles (Barnes and Hu, 2016).

Compared with the vertical angle, the wider angle range not only resulted in more information on the crop canopy, but also expanded the application range of the sensor, increasing overall efficiency. In addition to determining the best observation angle, it is also important to comprehensively model data from different angles to increase application accuracy (Guo et al., 2018; He et al., 2018). The WEI constructed in this study provided high estimation accuracy (*R*^2^ = 0.623) within a range of −20–10°, and compared with the optimal angle (−10°), the estimation accuracy of WEI within a range of −20–10° decreased by only 1.93%, while the RMSE value increased by only 4.71%. These results suggest that the WEI reduces the sensitivity to observation angles within the range of −20–10°, helping establish a more unified monitoring model, and increasing the efficiency and applicability of portable monitors in the field. However, despite these findings, this experiment was carried out using only three winter wheat varieties under two ecological conditions, and therefore, further analysis of the monitoring model in other regions, and with different crop types and varieties is required.

## Conclusion

Real-time monitoring of crop water use is of great significance in improving crop irrigation management and guiding water-saving agricultural production. Based on the WUE, this study adopted a combined vegetation index method, using NDDAig/FWBI to effectively determine the WUE of winter wheat leaves reflecting a new index named the WEI. The WEI performed better than other common vegetation indexes at 13 observation angles, with the most suitable observation range falling between −20 and 10°. Within this range, a unified estimation model was established with reduced dependency on observation angle limitations. These findings provide a basis for the selection of varieties with a high WUE as well as supporting water-saving cultivation management.

## Data Availability Statement

The raw data supporting the conclusions of this article will be made available by the authors, without undue reservation.

## Author Contributions

H-YZ, C-YW and WF conceived the research concept; H-YZ, M-RL, LS and XL performed the experiments; H-YZ, C-YW wrote the paper; Z-HF and W-DL contributed to the results analysis and discussion.

### Conflict of Interest

The authors declare that the research was conducted in the absence of any commercial or financial relationships that could be construed as a potential conflict of interest.
